# Active Herpes Zoster Mimicking Worsening of Axillary Lymph Node Metastases of Breast Cancer after Chemotherapy on 18F-Fluorodeoxyglucose Positron-Emission Tomography/Computed Tomography

**DOI:** 10.3390/diagnostics11061085

**Published:** 2021-06-14

**Authors:** Tomoyuki Fujioka, Kota Yokoyama, Mio Mori, Yuka Yashima, Emi Yamaga, Kazunori Kubota, Jun Oyama, Goshi Oda, Tsuyoshi Nakagawa, Ukihide Tateishi

**Affiliations:** 1 Department of Diagnostic Radiology, Tokyo Medical and Dental University, 1-5-45, Yushima, Bunkyo-ku, Tokyo 113-8519, Japan; fjokmrad@tmd.ac.jp (T.F.); kota1986ky@yahoo.co.jp (K.Y.); 11.ruby.89@gmail.com (Y.Y.); ymgdrnm@tmd.ac.jp (E.Y.); ooymmrad@tmd.ac.jp (J.O.); ttisdrnm@tmd.ac.jp (U.T.); 2 Department of Radiology, Dokkyo Medical University Saitama Medical Center, Saitama 343-8555, Japan; kubotard@dokkyomed.ac.jp; 3 Department of Surgery, Breast Surgery, Tokyo Medical and Dental University, Tokyo 113-8519, Japan; odasrg2@tmd.ac.jp (G.O.); nakagawa.srg2@tmd.ac.jp (T.N.)

**Keywords:** herpes zoster, PET/CT, axillary lymph node metastases, breast cancer, lymphadenopathy

## Abstract

A woman in her 60s presented to our hospital with a left breast mass that was diagnosed as breast cancer. 18F-Fluorodeoxyglucose positron-emission tomography/computed tomography (18F-FDG PET/CT) revealed intense, hot uptake in the cancerous mass and left axillary lymph node metastasis. After chemotherapy, another PET/CT scan was performed. Although the mass and left axillary lymph nodes shrank and FDG uptake decreased, enlarged lymph nodes with high FDG uptake appeared in the right axilla. The patient had a painful vesicular eruption on the front to the back of the right upper hemithorax, which was diagnosed as active herpes zoster. Active herpes zoster mimics a worsening axillary lymph node metastasis on the PET/CT scan.

A woman in her 60s with no significant medical history presented to our hospital with a left breast mass. Breast ultrasonography revealed left breast mass and enlarged left axillary lymph nodes, which were diagnosed as triple-negative breast cancer (invasive ductal carcinoma) and axillary lymph node metastasis by ultrasonography-guided biopsy, respectively. 18F-Fluorodeoxyglucose positron-emission tomography/computed tomography (18F-FDG PET/CT) was performed using a digital PET/CT system (Cartesion Prime, Canon Medical Systems, Tochigi, Japan) with the following parameters: dose of 18F-FDG, 3.7 MBq/kg (0.1 mCi/kg); emission time per bed, 2 min; bed position, 9–10; slice thickness, 4.08 mm; and matrix, 144 × 144. The scan revealed an intense hot uptake in the left breast mass (SUVmax, 10.0; a, b: blue arrows) and left axillary lymph nodes (SUVmax, 13.7; a, c, d: yellow arrows). No other areas of uptake indicated a suspected distant metastasis (a).

One week after chemotherapy (11 cycles of adjuvant paclitaxel after four cycles of adjuvant doxorubicin/cyclophosphamide), another PET/CT scan was performed. Although the left breast cancer (SUVmax, 2.5; d, e: blue arrows) and left axillary lymph nodes (SUVmax, 5.8; d, g: yellow arrows) shrank and the FDG uptake decreased, enlarged lymph nodes with high FDG uptake appeared in the right axilla (SUVmax, 23.4; d, f, g: red arrows). Two days before the PET/CT scan, the patient had a painful vesicular eruption on the front (h) to back (i) of the right upper hemithorax, which was diagnosed as active herpes zoster. PET/CT images were reviewed, and thickening of the skin on the right upper hemithorax with mild FDG uptake was found (f, g: white arrows). Ultrasound-guided fine needle aspiration cytology was performed on the right axillary lymph node, which was found to be benign (class I).

The patient received antiviral medication, which subsequently resolved her symptoms. She then underwent left total mastectomy and left axillary lymph node dissection. Three months after the surgery, the patient was alive and recurrence-free.

Herpes zoster is a reactivated varicella-zoster virus infection that causes a typical painful vesicular eruption on the dermatome. The latent virus increases and reactivates if cell-mediated immunity is compromised because of chronic disease, immune abnormalities, administration of anticancer drugs, immunosuppression, trauma, or stress [1]. Inflammation occurs when reactivated virus causes damage to the skin and nerves. The hypermetabolic activity of the inflammatory cells caused an increase in FDG accumulation [2]. Active herpes zoster mimics a worsening axillary lymph node metastasis on PET/CT [1,3,4,5]. When a PET/CT scan shows an unexpected abnormal uptake in the axillary lymph nodes, the radiologist should determine if the patient has a painful vesicular eruption on the ipsilateral upper hemithorax. Ultrasound-guided puncture aspiration cytology can help in the diagnosis of axillary lymph nodes [6].

**Figure 1:**
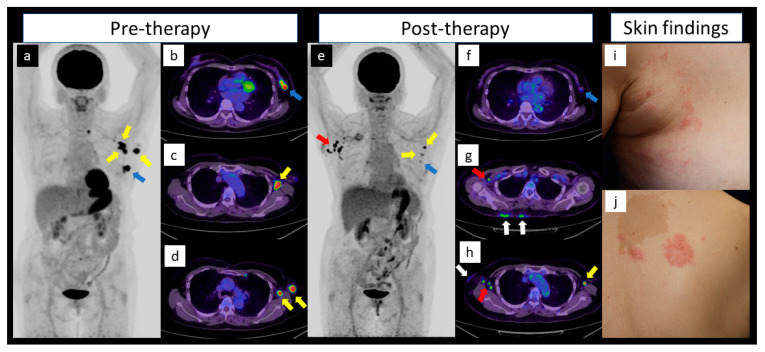
(**a**) Maximum-intensity projection (MIP) and (**b**–**d**) transaxial 18F-Fluorodeoxyglucose positron-emission tomography/computed tomography (18F-FDG PET/CT) images before therapy revealed (**a**,**b**: blue arrows) intense, hot uptake in the left breast mass indicating cancer, and (**a**,**c**,**d**: yellow arrows) left axillary lymph nodes indicating metastasis. (**a**) No other areas of uptake indicated distant metastasis. (**e**) MIP and (**f**–**h**) transaxial images of 18F-FDG PET/CT after therapy showed shrinking and decreased uptake in the (**e**,**f**: blue arrows) cancerous mass and (**e**,**h**: yellow arrows) left axillary lymph node metastasis, whereas (**e**,**g**,**h**: red arrows) enlarged lymph nodes with high FDG uptake appeared in the right axilla. (**g**,**h**: white arrows) Thickening of the skin on the right upper hemithorax with mild FDG uptake was also observed. The patient had painful vesicular eruption on the (**i**) front to (**j**) back of the right upper hemithorax, which was diagnosed as active herpes zoster.

## Data Availability

All available data are presented within the article or are available on request from the corresponding author.
